# *Limosilactobacillus fermentum* ING8, a Potential Multifunctional Non-Starter Strain with Relevant Technological Properties and Antimicrobial Activity

**DOI:** 10.3390/foods11050703

**Published:** 2022-02-26

**Authors:** Shadi Pakroo, Armin Tarrah, Rohit Takur, Manyu Wu, Viviana Corich, Alessio Giacomini

**Affiliations:** Department of Agronomy Food Natural Resources Animal and Environment (DAFNAE), University of Padova, Viale dell’Università 16, Legnaro, 35020 Padova, Italy; shadi.pakroo@phd.unipd.it (S.P.); rohit.takur@studenti.unipd.it (R.T.); manyu.wu@studenti.unipd.it (M.W.); alessio.giacomini@unipd.it (A.G.)

**Keywords:** exopolysaccharides, galactose, dahi, *Limosilactobacillus fermentum*

## Abstract

Lactic acid bacteria (LAB) have gained particular attention among different exopolysaccharide-producing microorganisms due to their safety status and effects on human health and food production. Exopolysaccharide-producing LAB play a crucial role in different ways, such as improving texture, mouthfeel, controlling viscosity, and for low-calorie food production. In this study, we isolated a multifunctional strain with good exopolysaccharide production properties. *Limosilactobacillus fermentum* ING8 was isolated from an Indian traditional fermented milk (Dahi) and evaluated for its safety, enzymatic activity, NaCl resistance and temperature tolerance, milk coagulation, and storage stability. Finally, the complete genome of this strain was sequenced and subjected to safety in silico evaluation and genomic analysis. The results revealed that *L. fermentum* ING8 possesses relevant technological properties, such as exopolysaccharide production, antimicrobial activity, and galactose utilization. Besides, this strain showed very high stability to storage conditions at refrigeration temperature. In addition, the genomic analysis did not evidence any possible deleterious elements, such as acquired antibiotic resistance genes, virulence genes, or hemolysis-related genes. However, all structural genes related to the galactose operon and EPS production were detected. Therefore, *L. fermentum* ING8 can be considered a promising multifunctional bacterium to be proposed as non-starter in different types of dairy productions.

## 1. Introduction

“Dahi,” that means curd, is an Indian traditional fermented milk prepared from either cow, buffalo, or goat milk, and it is considered one of the oldest consumed dairy products in India, Pakistan, Nepal, and Bangladesh [1]. Due to the unknown composition of microbial starter cultures and the uncontrolled fermentation process, traditional fermented foods can have wide variability in their microbiological, nutritional, and functional properties. The Dahi made from buffalo milk is usually more acidic and contains a higher amount of protein, calcium, fat, and lactose, providing more energy than those made from cow milk [2]. In addition, it is enriched with iron, phosphorus, vitamin A, and natural antioxidants, while the cholesterol content is comparatively lower than cow milk [3]. *Limosilactobacillus fermentum* is a lactic acid bacterium (LAB), Gram-positive, non-spore-forming, rod-shaped that produces organic acid from the fermentation of carbohydrates [4]. *L. fermentum* can also inhibit the growth of food-borne pathogens in food products [5], and it possesses the “generally recognized as safe” (GRAS), [6]. Foods obtained from fermentation by *L. fermentum* usually possess palatability, high sensory quality, texture, stability, and nutritional properties [7].

The main technological aspect which defines LAB is its capability to produce lactic acid by fermenting different sugars [8]. Among carbohydrates, galactose accumulation in dairy products following lactose fermentation is problematic and challenging. The accumulation of galactose can cause a browning effect on heat-treated cheeses such as Asiago, Parmigiano Reggiano, and Grana Padano in different foods and can damage their appearance [9,10]. In addition, galactose accumulation in dairy products can lead to toxic effects on people suffering from galactosemia [11]. Therefore, the use of starter cultures capable of utilizing galactose could be advantageous for the dairy industry. In addition to the production of lactic acid, LAB is used in the food industry for other relevant properties, such as their proteolytic and lipolytic activity, ability to synthesize a wide range of compounds such as organic acids, peptides, antimicrobial agents, aromatic compounds by the metabolism of citrate, and production of exopolysaccharides (EPS) [12]. EPS plays a crucial role in food production by improving texture in low-calories food products and in dietary fibers products [13]. In addition, they can be useful to human health by providing beneficial effects such as cholesterol-lowering activity and immune-stimulating properties [14]. In this study, Dahi samples made from buffalo milk were collected from different household markets in India. The safety and technological properties of the newly isolated LAB strains were investigated by phenotypical approaches. Finally, the complete genome of the promising strain *L. fermentum* ING8 as a strong lactose/galactose utilizer with good EPS production was sequenced for further in silico evaluation and genomic analysis.

## 2. Materials and Methods

### 2.1. Sample Collection and Isolation of New LAB Strains

Eight Dahi samples were collected from different villages of the Gadwal District Telangana state (India) during summer 2018. All samples were transferred into sterile plastic tubes and stored at 4 °C. For LAB isolation, 10 g of each sample were homogenized with 90 mL of sterile phosphate-buffered saline (PBS; NaCl 8.0 g/L, KCl 0.2 g/L, Na_2_HPO_4_ 1.44 g/L, KH_2_PO_4_ 0.24 g/L, pH 7.4, Sigma-Aldrich, Saint Louis, MO, USA). Then, decimal dilutions of the solution were prepared to up to 10^−9^ and all were inoculated on MRS (De Man Rogosa Sharp, Sigma-Aldrich) agar using the pour plate method. Plates were incubated in anaerobic jars at 30 °C for 72 h. Finally, one representative colony was taken from each sample unless different colony morphology was detected. All the isolates were examined for preliminary phenotypical tests, i.e., Gram staining, microscopic morphology, catalase, and oxidase [15,16]. The stock cultures were prepared in MRS broth inoculated with 25% (*v/v*) glycerol (Sigma-Aldrich) and kept at −80 °C until further use.

### 2.2. DNA Extraction and RAPD-PCR Analysis

DNA extraction was conducted using a bacterial lysate protocol according to Tarrah et al. [17] with slight modifications. Briefly, 5 mL of overnight bacterial cultures were washed 2 times with sterilized PBS and added to 50 µL of lysis buffer (NaOH 0.05 M + SDS 0.25%, Sigma-Aldrich). This solution was vortexed for 2 min and then incubated at 94 °C for 15 min in a thermal cycler (Bio-Rad Laboratories, Hercules, CA, USA). Finally, the supernatant was collected by centrifugation at 13,000× *g* for 10 min and diluted at 1:100 in ultrapure sterile water. The RAPD-PCR analysis was performed using primer M13 (5′ GAGGGTGGCGGTTCT 3′) according to the protocol by Andrighetto et al. [18]. RAPD-PCR profiles were analyzed using the software package Gel Compare Version 4.1 (Applied Maths, Kortrijk, Belgium) based on the Pearson product-moment correlation coefficient.

### 2.3. Lactose/Galactose Utilizers Selection 

A total of 10 mL of overnight bacterial cultures were centrifuged at 5000 rpm for 10 min; the pellets were washed twice using 5 mL of PBS and resuspended in 5 mL of the same solution. Lactose and galactose (Sigma-Aldrich) solutions (10% *w*/*v*) were prepared separately, filtered using 0.22 µm filters (Sigma-Aldrich) and added to MRS glucose-free medium to a final concentration of 1%. Finally, the media (MRS + 1% lactose/MRS + 1% galactose) were inoculated with 10^6^ (CFU/mL) overnight bacterial cultures and incubated at 37 °C for 24 h using 96-well microtiter plates (sigma SIAL0596, MO, USA) and a Tecan incubator/reader (spark 10 M, Tecan GmbH, Grödig. Austria). 

The optical density was measured for both lactose and galactose by reading the absorbance at 600 nm (OD600) every 30 min for 24 h. Blank and negative controls were inserted as well. The experiment was conducted using two biological and four technical replicates.

### 2.4. Exopolysaccharide Production and Quantification 

Overnight bacterial cultures were streaked on a modified MRS agar medium containing 10% sucrose and incubated at 30 °C for 3 days to allow EPS production. Strains generating slimy colonies were considered EPS producers [19]. EPS producer strains on MRS agar were selected for EPS quantification using MRS broth containing 10% sucrose. Overnight culture (1%) was used to inoculate MRS broth containing 10% sucrose and incubated at 30 °C for 3 days. Then the supernatant was collected by centrifugation at 5500 rpm for 20 min, and EPS was precipitated by using five volumes of cold 96% ethanol (Sigma-Aldrich) and incubating for 24 h. Finally, the non-purified EPS was collected by centrifugation at 4 °C (5500 rpm for 20 min), dried at 60 °C overnight, and the weight was recorded [20]. The experiment was conducted using 2 biological and 3 technical replicates.

### 2.5. Antimicrobial Activity Determination

A total of 10 µL of 24 h cultures were placed on 2 MRS agar plates and incubated overnight. After the incubation, in one of the plates, 2 µL of proteinase K (Sigma-Aldrich) solution (20 mg/mL, pH 8) were spotted close to the bacterial growth to determine the production of bacteriocin compounds and incubated for 1 h. Successively, a lawn of 4 mL of BHI soft agar (Sigma-Aldrich) containing 400 µL of an overnight culture of indicator strains, namely *Listeria monocytogenes* ATCC 19117, *Bacillus cereus* ATCC 11778, *Escherichia coli* APEC 18042/2 was poured on the top of the existing solid medium and plates were further incubated at 37 °C for 24 h. After incubation, plates were inspected for zones of indicator strains growth inhibition surrounding the colonies. The presence of inhibition haloes in the MRS plate and the absence of haloes for the same strain in the protease-containing plate indicate the proteinaceous origin of the antimicrobial compound [21]. 

### 2.6. Safety Assessments of Strain L. fermentum ING8

#### 2.6.1. Minimum Inhibitory Concentration (MIC)

The MIC test was performed in 96-well microtiter plates according to the broth microdilution method proposed by Wiegand et al. [22]. The following antibiotics were chosen, according to the European Food Safety Authority (EFSA) recommendation: vancomycin, kanamycin, ampicillin, tetracycline, erythromycin, chloramphenicol, streptomycin, gentamicin, and ciprofloxacin, Sigma-Aldrich). The antibiotics were dissolved in MRS plus ISO-Sensitest (Sigma-Aldrich) broth in 1:9 ratio, serially diluted in 96-microtiter plates, and further inoculated with bacterial cells (5 × 10^5^ CFU/mL). The test was performed in 3 replicates, and the MIC value was considered the concentration present in the first well with no visible growth.

#### 2.6.2. Hemolytic Activity Test

A total of 20 µL of overnight cultures were spotted on MRS plates containing 5% (*w*/*v*) sheep blood (Thermo Fisher Scientific, Waltham, Massachusetts, United States) and incubated at 37 °C for 48 h. Hemolytic activity was determined by a clear halo surrounding the colonies [23]. *Staphylococcus aureus* ATCC 6538 and *Lacticaseibacillus rhamnosus* GG were included as positive and negative controls, respectively. 

### 2.7. Technological Characterization of L. fermentum ING8

#### 2.7.1. Enzymatic Activities 

The amylolytic activity was determined using a 2-step procedure by first culturing the strain ING8 in a modified MRS broth (MRS with 0.25% starch instead of glucose, Sigma-Aldrich), incubated at 37 °C for 24 h. Subsequently, 10 µL of the cultured bacteria were spotted on a medium containing 0.5% meat peptone, 0.7% yeast extract, 0.2% NaCl, 2% starch and 1.5% agar (Sigma-Aldrich) on plates incubated anaerobically at 37 °C for 48 h. The amylolytic activity was determined by detecting clear zones around the colonies after staining with Lugol solution (Sigma-Aldrich).

The lipolytic activity was evaluated using a two-step procedure by culturing the bacteria in a modified MRS medium containing 1% olive oil and 1% Arabic gum (Sigma-Aldrich), and incubating at 37 °C for 24 h. Successively, 10 µL of subcultured bacteria were spotted on a medium containing 0.1% tryptone, 0.5% yeast extract, 0.05% NaCl, 0.1, 0.5, or 1% olive oil, 1% Arabic gum, and 1.5% agar (Sigma-Aldrich) and the plates were incubated anaerobically at 37 °C for 48 h. The lipolytic activity was evaluated by the detection of a clear zone around the colonies. 

Regarding the proteolytic activity, the bacterial strain was cultured in MRS broth incubated anaerobically at 37 °C for 24 h. After incubation, 30 μL of the supernatant were collected and spotted on a sterile paper disc, later placed over a medium containing 1% skim milk (Sigma-Aldrich) and 1.5% agar. Then plates were incubated anaerobically for 48 h at 37 °C. The clear zone around each disc was measured with a caliper to determine the level of proteolytic activity [24].

#### 2.7.2. NaCl Tolerance Test 

Strain ING8 was subcultured in MRS medium incubated at 37 °C for 24 h. After centrifugation, the pellet was washed 2 times with PBS and transferred to MRS supplemented with 2, 6, and 10% (*w*/*v*) NaCl, and incubated at 37 °C for 24 h. The growth ability was checked by reading the optical density (OD 600 nm) using 96-well microtiter plates and Tecan reader [19]. The experiment was conducted using 2 biological and 3 technical replicates.

#### 2.7.3. Growth at Different Temperatures

The ability of bacterial growth at different temperatures was conducted using the protocol published by Ribeiro et al. with slight modification [19]. The strain ING8 was cultured in MRS broth medium incubated at 30 °C, 37 °C, 45 °C, and 50 °C, respectively, for a period of 24 h. The growth was checked by reading the optical density (OD 600 nm) using 96-well microtiter plates and a Tecan reader. The experiment was conducted using two biological and three technical replicates.

#### 2.7.4. Acidification and Milk Coagulation

The acidification and milk coagulation capability of strain ING8 were evaluated using a modified protocol by Shangpliang et al. [1]. Briefly, the overnight culture of ING8 was centrifuged, the pellet washed 2 times using sterile PBS and resuspended in 5 mL of the same buffer. Later, 100 µL of this solution were used to inoculate 10 mL sterile of skimmed milk (10%) incubated at 30 °C. The pH value and coagulation status were determined after 3, 6, and 24 h. The experiment was conducted using 2 biological and 3 technical replicates.

#### 2.7.5. Viability during Storage

An overnight culture of ING8 was centrifuged, the pellet washed 2 times using sterile PBS, resuspended in 10 mL sterile skimmed milk (10%) and stored at 4 °C for 0, 7, 14, and 21 days [25]. At each time interval, an aliquot was taken, serially diluted, and plated on MRS agar to enumerate viable bacteria. The experiment was conducted using 3 technical replicates. 

### 2.8. Genome Sequencing, Assembly, and Annotation of L. fermentum ING8

According to the manufacturer’s instructions, genomic DNA from strain ING8 was extracted using the DNeasy PowerSoil Kit (Qiagen, Hilden, Germany). The quality of the extracted DNA was checked spectrophotometrically (Spark 10 M, Tecan). The genome sequencing was conducted using the paired-end sequencing technology with an Illumina MiSeq sequencer (Ramaciotti Centre for Genomics, Sydney, Australia). A Unicycler assembler was used to assemble the raw reads by using the PATRIC database server 3.6.12, setting the parameters on default [26]. The prediction and annotation of the assembled genome were carried out using the Rapid Annotation using Subsystems Technology (RAST) [27], and the graphical genome map of *L. fermentum* ING8 was constructed using the CGView (Circular Genome Viewer) [28] after scaffolding the assembled contigs using the medusa webserver [29] and the type strain *L. fermentum* strain DSM 20052 as the reference genome.

Genome stability and safety were assessed by studying the presence of virulence genes, plasmids and acquired antibiotic resistance genes within the genome of *L. fermentum* ING8 by using IslandViewer4, PlasmidFinder 2.0, and ResFinder 3.2 servers [30,31,32].

### 2.9. Statistical Analysis

Data were analyzed by one-way analysis of variance (ANOVA) using GraphPad Prism software (version 7, GraphPad Software, Inc., San Diego, CA, USA).

## 3. Results

### 3.1. Strains Isolation and RAPD-PCR Analysis

**Figure 1:**
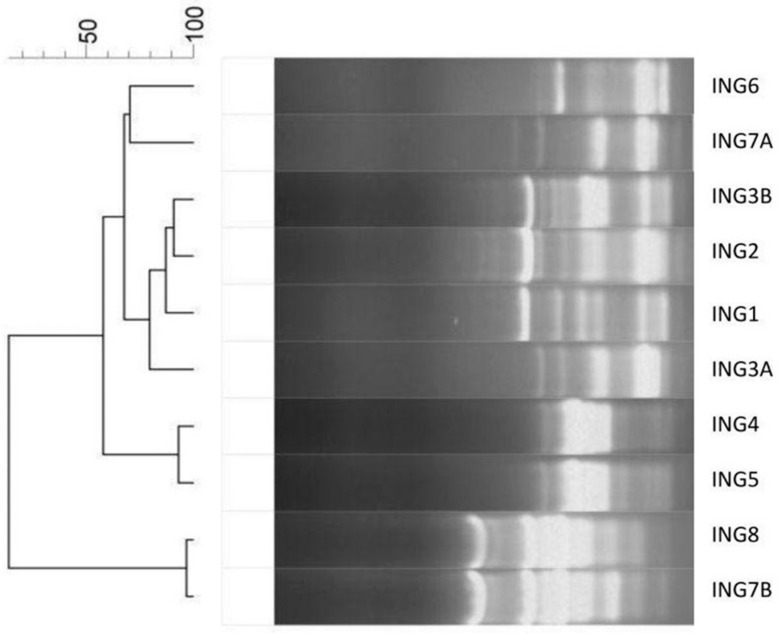
Cluster analysis of RAPD-PCR fingerprints of the isolates.

Isolates from 10 different colonies (ING1, ING2, ING3A, ING3B, ING4, ING5, ING6, ING7A, ING7B, ING8) were collected from MRS agar plates considering colony morphology. All isolates were rod-shaped, Gram-positive, catalase-negative, and oxidase-negative. A RAPD-PCR analysis was performed to compare the isolates based on their electrophoretic profiles (Figure 1). According to the UPGMA analysis performed by Gel Compare software, isolates ING7B with ING8 and ING4 with ING5 showed highly similar profiles. 

### 3.2. Lactose and Galactose Utilization 

**Figure 2:**
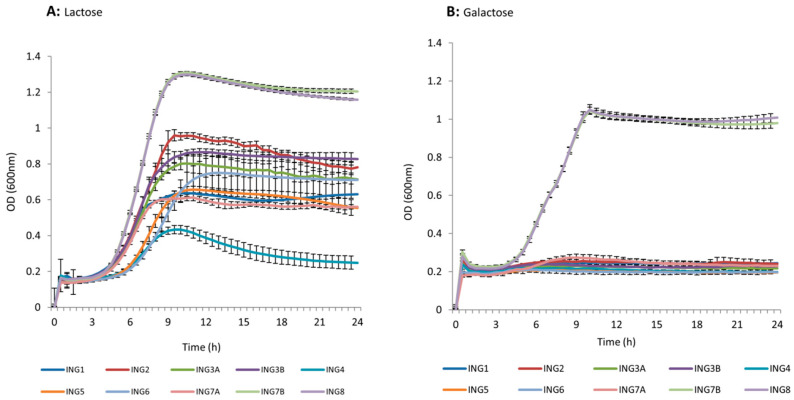
Growth curves of strains on lactose (**A**) and galactose (**B**).

All tested strains could grow on a medium containing lactose, except strain ING4, which revealed a poor growth after incubation (OD600 = 0.2 was considered the growth threshold based on negative controls), whereas isolates ING7B and ING8 showed excellent growth compared with the other strains (Figure 2A). On the other side, all bacteria failed to grow on MRS medium containing galactose as the sole energy source except for ING7B and ING8, which revealed a very good capability to utilize galactose (Figure 2B). Interestingly, isolates ING7B and ING8 overlapped entirely in galactose and lactose utilization dynamics, confirming the very high similarity detected by RAPD-PCR analysis, while ING4 and ING 5, which also showed very similar profiles, behaved very differently on lactose. For this reason, isolate ING7B was not considered for further studies.

### 3.3. Exopolysaccharide Production 

Among all the strains examined for EPS production, ING8 was the only one producing sticky colonies (4 mm diameter) after 3 days at 30 °C using MRS agar containing 10% sucrose. The quantification of EPS resulted in 400 ± 18 mg/L. 

### 3.4. Antimicrobial Activity Determination

All the strains were tested for antimicrobial activity against food-borne pathogenic bacteria. Among all, only strain ING8 produced a growth inhibition zone greater than 31 mm against Listeria monocytogenes ATCC 19117, Bacillus cereus ATCC 11778, and Escherichia coli APEC 18042/2 (Table 1). Indeed, the same haloes were also detected in the plates added with proteinase K, indicating the non-proteinaceous nature of such inhibitory activity. 

According to the results, strain ING8, due to its galactose utilization capability, antimicrobial activity, and high amount of EPS production, was selected for further safety and technological assessments.

### 3.5. Safety Assessments of L. fermentum ING8

The MIC for nine antibiotics, recommended by the European Food Safety Authority (EFSA), was evaluated for strain ING8. As a result, the strain was susceptible to tetracycline, erythromycin, ampicillin, chloramphenicol, gentamycin, streptomycin, and ciprofloxacin. However, it was resistant to kanamycin and vancomycin (Table 2). On the other side, strain ING8 did not reveal any hemolytic activity when spotted on MRS plates containing 5% (*w*/*v*) sheep blood. 

### 3.6. Technological Properties of L. fermentum ING8

**Figure 3:**
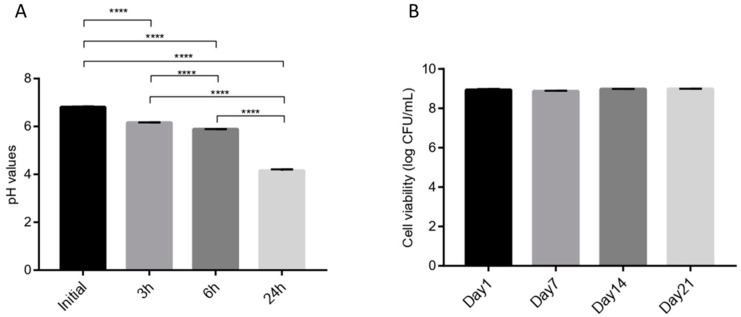
**(A**) pH reduction of *L. fermentum* ING8, (**B**) Survivability of *L. fermentum* ING8 cells at 4 °C for 0, 7, 14, and 21 days. The number of asterisks is used to indicate thelevel of confidence of the statistical analyses results: **** (*p* < 0.0001).

Proteolytic, lipolytic, and amylolytic activities assessments did not reveal the presence of any of these properties in strain ING8 (Table 3). Regarding NaCl tolerance, ING8 could grow well in MRS containing 2% and 6% NaCl, but it was not able to grow in the presence of 10% NaCl (Table 3). On the other hand, it showed the optimal capability to grow at 30 °C and 37 °C, and very good to 45 °C and 50 °C, with relatively good acidification capability (around pH 4) after 24 h (Figure 3A) and coagulation ability (after 24 h) tested in 10% sterile skimmed milk. Regarding the viability during 21 days of storage, we did not detect any reduction in viability of ING8 in 10% skimmed milk stored at 4 °C (p ≤ 0.05) (Figure 3B).

### 3.7. Genomic Analysis of L. fermentum ING8

**Figure 4:**
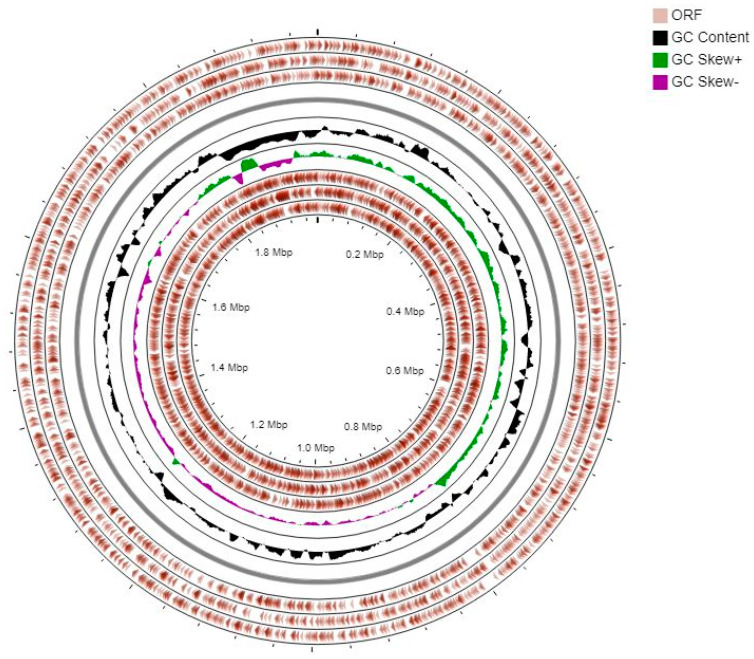
The circular graphical genome map of *L. fermentum* ING8, from outer to inner rings, ORF on the forward strand, GC content, GC skew, and ORF on the reverse strand.

**Figure 5:**
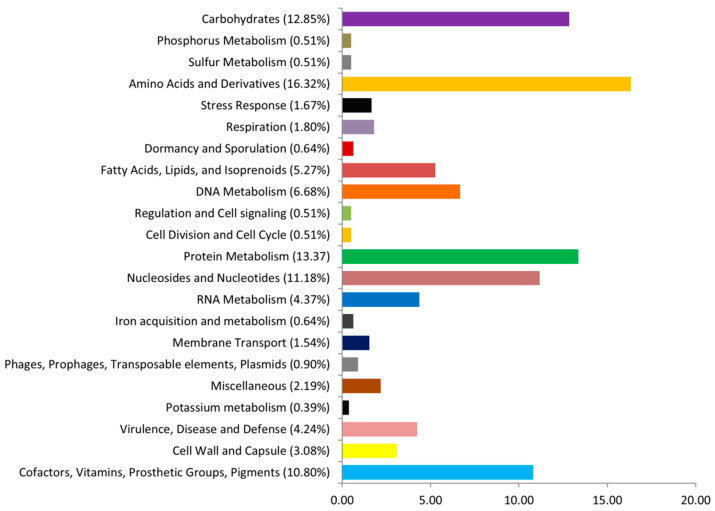
The overview of the RAST annotation and subsystems for *L. fermentum* ING8 genome.

The assembled genome of the EPS producer *L. fermentum* ING8 generated 25 scaffolds (158 contigs), giving a full genome size of 1.98 Mb with 51.3 GC% content and genome coverage of 51× (Table 4). The circular graphical genome map of *L. fermentum* ING8 is reported in Figure 4. The gene prediction conducted by RAST revealed a total of 2103 protein-coding sequences (CDSs) classified in 208 SEED subsystems. A total of 58 structural RNAs and 13 genes related to osmotic, oxidative, and detoxification stress responses were predicted after the annotation (Figure 5). Neither virulence-related genes nor acquired antibiotic resistance and plasmid sequences were detected after the annotation (Table 4). The largest section of the ING8 genome subsystem was dedicated to amino acids and derivatives (16.32%), followed by protein metabolisms (13.37%) and carbohydrate metabolism with 12.85%, respectively (Figure 5). Moreover, a deep search using the RAST search tool for detection of galactose consumption-related genes and EPS production-related genes within the genome of *L. fermentum* ING8 revealed the presence of the complete gal operon with all its structural genes (galK, galT, galE, galM, galR, and galA) and of EPS production-related genes (epsB, epsC, epsD, and epsE).

## 4. Discussion

LAB has gained particular attention among different EPS-producing microbes due to their application in the food industry and related health issues. This group of bacteria plays a crucial role in food production in different ways by controlling viscosity, improving texture, improving mouthfeel, freeze-thaw stability, being used in low-calorie food products, dietary fibers products, and so on [33,34]. On the other hand, LAB can be helpful to human health by providing some beneficial effects such as anti-cancer, anti-ulcer, antioxidant potential, cholesterol-lowering activity, and immune-stimulating properties [35]. *L. fermentum* strains are used in various food products to increase their preservation, sensory characteristics, nutritional value, and other properties [7]. This species is also naturally present in diverse cheeses due to its technological properties, such as exopolysaccharide production, to improve textural and organoleptic properties [7,36]. EPS producing bacteria can also reduce syneresis of fermented milk due to the water-holding properties of EPS, which can lead to a higher quality of low-fat dairy products [7]. Many studies have been conducted on the identification of EPS producing LAB from dairy environments due to its importance in food production [37]. The amount of EPS produced by LAB is strain-dependent [37]. In a study by Fukuda et al. [38], *L. fermentum* TDS030603 was found to produce a highly viscous EPS molecule with a yield of 100 mg/L. In another study reported by Wei et al. [39], the EPS amount produced by *L. fermentum* YL-11 was estimated at around 84.5 mg/L, and it indicated cytotoxic activity against HT-29 and Caco-2 colon related carcinogenic cells. In a recent study by Butorac et al., *L. fermentum* D12 was found to produce up to 200 mg/L of EPS, which could lead to strain functionality such as antimicrobial activity against the pathogens [40]. EPS are long chains of different repeating units of sugars such as rhamnose, galactose, and glucose [41]. Regarding their structure, EPS are very different in molecular mass, molecular size, charge, and, consequently, rheological properties. EPSs from LAB are highly variable polymers that can be classified following their monomer compositions and grouped into homo-exopolysaccharides (HoPS) or hetero-exopolysaccharides (HePS) [42]. Biosynthesis of EPSs by LAB is a complex process that requires a large number of enzymes and proteins. Generally, it has been categorized into four steps initiating from sugar transport into the cytoplasm, synthesis of 1P-sugar, polymerization of repeating units precursors, and EPS transport outside the cell. The genes involved in the production of EPS can be located both on plasmids or on chromosomal DNA [43].

On the other side, carbohydrates, in general, are the primary energy source for fermentation and acidification by LAB. For the transport within the cell, different systems exist in bacteria. The main system is the PTS (Phospho Transferase System), but there is an alternative pathway for galactose. When galactose is imported into the cell by a specific permease, it uses the Leloir pathway to produce lactic acid as the final product. In galactose-positive strains, this sugar is metabolized by the Leloir pathway, which includes four different enzymes, namely galactose mutarotase (GalM), galactokinase (GalK), galactose-1-phosphate uridylyltransferase (GalT), and UDP-glucose 4-epimerase (GalE) [44]. However, most LAB strains are galactose negative due to the weak induction of *gal* operon. Galactose accumulation in many kinds of cheese such as Asiago, Parmigiano Reggiano, Swiss, and Grana has been reported and resulted in discoloration and browning negative effects [9,10].

Galactose accumulation can also be life-threatening due to the toxic effects on people suffering from galactosemia, a genetic disorder in humans [11]. Therefore, galactose-positive strains can be useful for reducing such technological and health problems. In Parmigiano Reggiano and Grana Padano cheese production, 10–20% of *L. fermentum* is used as starter culture along with *Streptococcus thermophilus* and thermophilic lactobacilli [36]. The availability of *L. fermentum* strains with a high level of galactose utilization and able to survive during prolonged storage periods can be beneficial since the problems mentioned above can frequently happen in long ripened cheeses.

Some LAB strains produce different antibacterial peptides named bacteriocins, which could inhibit other bacteria species, including pathogens [45]. Indeed, *L. fermentum* ING8 could contrast the growth of all indicator strains to a great extent, most probably because of organic acid production and pH lowering capability. 

Other technological properties such as proteolytic, lipolytic, and amylolytic activities of LAB can positively affect the texture and flavor of the products. In addition, starter cultures are usually exposed to some harsh conditions such as high temperatures or high NaCl concentration, depending on the type of cheese, which can cause early autolysis of starter cultures due to osmotic stress [46]. 

Finally, microbes that are used to produce food should be safe. They should come from the qualified list established by EFSA according to their taxonomy and very long periods of safe use and must be examined for the presence of acquired and transmissible antibiotic resistance genes within their genomes as well as hemolytic-related genes [23]. Therefore, the genome sequencing of new strains has become mandatory to precisely determine their taxonomy and retrieve all safety-related information present inside the genome [47,48]. 

## 5. Conclusions

In this study, we have isolated and studied *L. fermentum* ING8, a strain capable of galactose utilization with antimicrobial activity and good EPS production, which, interestingly, did not show any viability loss during 21 days of storage at refrigeration temperature. Besides, *L. fermentum* ING8 revealed relatively good tolerance to high temperatures and high concentrations of NaCl. On the other side, the deep genomic analysis of this strain did not reveal any possible deleterious characteristics such as the presence of virulence genes, acquired antibiotic resistance genes, or hemolytic-related traits. For these reasons, this strain has a good potential to be used as a multifunctional non-starter culture to produce different dairy products.

## Figures and Tables

**Table 1:** Antimicrobial activity by using spot on lawn technique.

	ING 1	ING 2	ING 3A	ING 3B	ING 4	ING 5	ING 6	ING 7A	ING 8
*L. monocytogenes* ATCC19117	+	+	+++	+	++	+	+	+	++++
*B. cereus* ATCC 11778	++	+	+	+	+	+	+	+	++++
*E. coli* APEC18042/2	+	+	+	+	++	+++	+	+	++++

Inhibition diagonal (mm) diameter ≤ 12 = (+); 13 ≤ diameter ≤ 20 = (++); 21 ≤ diameter ≤ 30 = (+++); diameter ≥ 31 = (++++).

**Table 2:** MIC values for human and veterinary relevant antibiotics. Breakpoints (μg/mL) suggested by EFSA are reported in parentheses.

Antibiotics									
Strain/Antibiotics	chloramphenicol	Gentamicin	tetracycline	erythromycin	ampicillin	streptomycin	vancomycin	Ciprofloxacin	Kanamycin
*L. fermentum* ING8	4 (4)	8 (16)	<0.125 (8)	0.5 (1)	0.5 (2)	16 (64)	>64 (n.r.) **	4 (n.r.)	32 (32)

Strains with MIC higher than the breakpoint (in bold) are considered resistant. ** n.r. not required by EFSA.

**Table 3:** Technological properties (growth at different temperatures, resistance to different NaCl concentrations, proteolytic, lipolytic, and amylolytic activity) of strain *L. fermentum* ING8.

	Different Temperatures (°C) *				Different NaCl (%) **						
	30	37	45	50	0	2	6	10	Proteolytic activity ***	Lipolytic activity ***	Amylolytic activity ***
*L. fermentum* ING8	+++	+++	++	++	+++	+++	++	-	-	-	-

* Growth measured by spectrophotometric method: OD ≤ 0.05 = (-); OD 0.05 ≤ 0.1 = (+); OD ≤ 0.5 = (++); OD > 0.5 = (+++). ** Growth measured by spectrophotometric method: OD ≤ 0.05 = (-); OD 0.05 ≤ 0.1 = (+); OD ≤ 0.5 = (++); OD > 0.5 = (+++). *** positive result (+), negative result (-).

**Table 4:** Genome features of *L. fermentum* ING8.

Feature	Value
Genome size	1,981,384
G + C content (%)	51.3
Contig N50	41442
Contig L50	17
Number of contigs	158
Number of Protein Coding Sequences (CDSs)	2103
Number of tRNAs	57
Number of rRNAs (5S, 16S, 23S)	3
Number of genes related to plasmid and acquired antibiotic-resistant gene	0
Number of genes related to Virulence, Disease, and Defense	0

## Data Availability

*L. fermentum* ING8 is part of the Department of Agronomy Food Natural Resources Animals and Environment collection, Padua University, Italy. The Whole Genome Shotgun project of *L. fermentum* ING8 has been deposited at DDBJ/ENA/GenBank under the accession JAJOHX000000000. The version described in this paper is version JAJOHX010000000.
